# Direct optogenetic stimulation of smooth muscle cells to control gastric contractility

**DOI:** 10.7150/thno.53883

**Published:** 2021-03-20

**Authors:** Markus Vogt, Benjamin Schulz, Ahmed Wagdi, Jan Lebert, Gijsbert J. van Belle, Jan Christoph, Tobias Bruegmann, Robert Patejdl

**Affiliations:** 1 Institute of Cardiovascular Physiology, University Medical Center Göttingen, Humboldtallee 23, 37073 Göttingen, Germany.; 2 Oscar Langendorff Institute for Physiology, University of Rostock, Germany.; 3 Department of Cardiology and Pneumology, University Medical Center Göttingen, Robert-Koch-Str. 42a-Heart Research Building, 37075 Göttingen, Germany.; 4 Current address: Cardiovascular Research Institute, University of California, San Francisco, 555 Mission Bay Blvd South, San Francisco, CA 94158, USA.

**Keywords:** optogenetics, gastric motility, gastroparesis, smooth muscle, channelrhodopsin

## Abstract

**Rationale:** Antral peristalsis is responsible for gastric emptying. Its failure is called gastroparesis and often caused by dysfunction of enteric neurons and interstitial cells of Cajal (ICC). Current treatment options, including gastric electrical stimulation, are non-satisfying and may improve symptoms but commonly fail to restore gastric emptying. Herein, we explore direct optogenetic stimulation of smooth muscle cells (SMC) via the light-gated non-selective cation channel Channelrhodopsin2 (ChR2) to control gastric motor function.

**Methods:** We used a transgenic mouse model expressing ChR2 in fusion with eYFP under the control of the chicken-β-actin promoter. We performed patch clamp experiments to quantify light-induced currents in isolated SMC, Ca^2+^ imaging and isometric force measurements of antral smooth muscle strips as well as pressure recordings of intact stomachs to evaluate contractile responses. Light-induced propulsion of gastric contents from the isolated stomach preparation was quantified in video recordings. We furthermore tested optogenetic stimulation in a gastroparesis model induced by neuronal- and ICC-specific damage through methylene blue photo-toxicity.

**Results:** In the stomachs, eYFP signals were restricted to SMC in which blue light (460 nm) induced inward currents typical for ChR2. These depolarizing currents led to contractions in antral smooth muscle strips that were stronger than those triggered by supramaximal electrical field stimulation and comparable to those evoked by global depolarization with high K^+^ concentration. In the intact stomach, panoramic illumination efficiently increased intragastric pressure achieving 239±46% (n=6) of the pressure induced by electrical field stimulation and triggered gastric transport. Within the gastroparesis model, electric field stimulation completely failed but light still efficiently generated pressure waves.

**Conclusions:** We demonstrate direct optogenetic stimulation of SMC to control gastric contractility. This completely new approach could allow for the restoration of motility in gastroparesis in the future.

## Introduction

Antral peristalsis is a prerequisite for effective gastric emptying and is generated by local contractions of the tunica muscularis which are coordinated and initiated by the electric slow waves within the interstitial cells of Cajal (ICC) and modulated by the enteric nervous system 1,2. While propagating along the longitudinal gastric axis, ICC spread depolarizing currents to adjacent smooth muscle cells (SMC). Thereby the membrane potential of SMC is driven electrotonically towards the activation threshold of L-type voltage dependent Ca^2+^-channels (VDCC) which ultimately gives rise to the Ca^2+^-entry and triggers SMC-contraction 3-5. Theoretically, alterations at any of these steps would interfere with gastric emptying. In fact, morphological and functional evidence suggests that it is by far most often the dysfunction of ICC and neurons that underlies impaired gastric emptying in the absence of mechanical obstruction, which is the clinical definition of gastroparesis 6-9. Although estimates on the prevalence and burden of gastroparesis vary widely between studies, it is undoubtedly a frequent and disabling chronic medical condition 10-12. Besides alleviating symptoms, most therapeutic strategies in gastroparesis intend to restore gastric emptying. Unfortunately, many have proven to be largely non-effective in achieving this goal 13. Surgical options like transpyloric stents, pylorotomy or gastrectomy may ameliorate some symptoms, but inevitably curtail the stomach's roles as a digestive organ and reservoir with the risk of severe secondary complications 14-17.

Various different pharmacological treatment strategies have been developed and used for the treatment of gastroparesis. Among others, receptors for dopamine, serotonin or ghrelin have been targeted with very variable and in many cases insufficient results regarding subjective symptoms and gastric emptying, but nevertheless with rather prevalent neurologic or cardiovascular side effects 18-20. An explanation for the disappointing effects on the motility itself may be that it is evidently not possible to specifically target SMC in the abovementioned selective and coordinated manner using systemic administration of drugs, no matter how specific and effective a substance may be in stimulating gastric SMC in general. Studies on gastric electrical stimulation for gastroparesis have tested various paradigms, i.e. stimulation with low frequencies and high energy targeting SMC directly on the one hand versus high-frequency stimulation with low energy modulating the afferent nerval signaling on the other. Unfortunately, high energetic electrical pulses cause discomfort and pain as well as co-stimulation of adjacent tissues including the diaphragm and are thus not tolerated by patients. Electrical field stimulation (EFS) approaching intramural sensory neurons with low energetic pulses failed to imitate physiological slow waves and improved patient's symptom scores only in first open-label trials 21-23. These limitations have prevented the widespread use of gastric electrical stimulation for decades.

Direct optogenetic stimulation of gastric smooth muscle would avoid these shortcomings of all current treatment strategies: Optogenetics allows to control cells in living tissues by stimulation with light after genetic modification of the desired cell type to express light sensitive proteins 24. Channelrhodopsin2 (ChR2) is a light-gated channel conducting non-selectively cations upon illumination with blue light and allows to control the membrane potential with high spatial and temporal resolution 25. In clear contrast to EFS, optogenetic stimulation can be performed cell-specific: If ChR2 is only expressed selectively in SMC, these alone will be affected by the illumination whereas other cell types such as nociceptive neurons in close proximity are not excited 26.

However, the use of optogenetic stimulation within the digestive tract has been restricted to neuronal stimulation of the colon 27-30. Such indirect stimulation may not or only minimally enhance gastric emptying in cases of gastroparesis due to the non-functional enteric nervous system as well as the depletion of ICC. Considering that the most frequent primary diseases underlying the pathological alterations of ICC and neuronal networks are progressive by nature (e.g. diabetes or Parkinson's disease), it seems obvious that treatments targeting these cells are at risk to become less effective over time or be non-effective at all.

In striated muscle 31, 32 and neurons 33-35, ChR2-mediated optogenetic stimulation has been shown to be an efficient and reliable trigger for action potentials. However, the threshold for excitation in these tissues is rather low due to their prominent fast sodium currents, whereas triggering spike-potentials in gastric smooth muscle relies on the opening of L-type VDCC which require stronger and more sustained depolarizations 36. Furthermore, since gastric SMC form a well-coupled syncytium (“single-unit smooth muscle”), considerable current loss from the illuminated cells to their non-depolarized surrounding has to be expected 37, 38. This makes ChR2-mediated optogenetic stimulation of intestine and stomach SMC more challenging than that of “multi-unit”-tissues as arterial smooth muscle, where effective light stimulation has been demonstrated recently 39.

The central question remains whether light-induced depolarization mediated by ChR2 is able to effectively trigger gastric contractions under physiologic conditions or even in stomachs with non-functional neuronal and ICC networks. Herein, we explore the feasibility of optogenetic gastric pacemaking in mice by direct excitation of SMC via the use of the light-sensitive protein ChR2 in single SMC, muscle tissue as well as whole-organs.

## Methods

### Transgenic animal model

All experiments were in accordance to the European Guideline for animal experiments 2010/63/EU Studies. Transgenic animals expressed ChR2 (H134R) 40 and eYFP under control of the chicken-β-actin promoter and were backcrossed at least 10 generations on a CD1 genetic background 31. 18 female and 18 male transgenic mice (21±2 weeks) and 13 female and 13 male CD-1 wildtype mice (23±4 weeks) were used. Mice were killed by cervical dislocation and the explanted stomachs controlled for ChR2/eYFP fluorescence signals.

### Isolation of single smooth muscle cells

Stomachs were placed in ice-cold Ca^2+^-free-Tyrode solution (Table 1). Muscle strips were carefully detached from the underlying mucosal layer and incubated at 4 °C for 30 min. Subsequently, strips were cut into small pieces and incubated in Ca^2+^-free-Tyrode solution supplemented with (in mg/ml): Papain 1 (LS003126, Worthington, USA), Trypsin Inhibitor 1 (T9128, Sigma-Aldrich, Germany), Blend Collagenase 1 (C8051, Sigma-Aldrich), Collagenase IV-S 1 (C1889, Sigma-Aldrich), Elastase 0.4 (20929.01, Serva, Germany) and Albumin Fraction V 10 (8076.2, Roth, Germany) for 30 min at 37 °C. Afterwards, enzyme-containing supernatant was carefully removed and muscle pieces were resuspended in Ca^2+^-Tyrode solution and filtered through a 200 µm pluriStrainer® (pluriSelect, Germany). Cells were seeded on glass plates coated with 500 µM fibronectin (F1141-5MG, Sigma-Aldrich), stored at 4 °C and used within 8 h.

### Patch clamp

Patch clamp experiments were performed in whole cell configuration with freshly pulled glass pipettes (3-5 MΩ) at room temperature using an inverted IX73 microscope equipped with a LUCPLFLN20XPH/0,45 objective (Olympus, Japan). Data was recorded at 10 kHz with an EPC-10 USB amplifier and the PatchMaster software (Heka, Germany). Illumination was performed with a LEDHub equipped with a blue LED (460 nm/3000 mW, Omicron, Germany). Light intensity was calibrated with the S170C sensor and Powermeter (Thorlabs, Germany).

Light-induced currents were characterized at -60 mV by subtracting the mean of the 400 ms before illumination from the maximum inward current (“peak current”) or the mean of the 250 to 750 ms of illumination (“steady state current”). For the current-voltage relation, we analyzed the mean of steady state currents during illumination at holding potentials from -100 mV to +60 mV increased in 10 mV steps. For analysis of the membrane resistance before and after the complete illumination protocol, 200 ms long ramps from -100 mV to +60 mV were performed at least five times and the change in current amplitude between -90 mV to -70 mV was averaged. Using determined values of voltage and current changes, membrane resistance was calculated according to Ohm's law.

Voltage-gated outward directed currents were evoked by voltage steps from -80 mV to +50 mV increased in 10 mV steps and quantified as maximal currents subtracted by the mean current of the last 800 ms of the respective voltage steps. Only cells that showed outward currents upon depolarization, defined as all voltage steps above -20 mV, indicated by having the maximal current amplitude within the first 500 ms of the 4 s long stimulation, were taken into consideration.

### Force measurements of antral smooth muscle strips

Longitudinal antral smooth muscle strips were excised from stomachs and kept in cooled preparation buffer. They were tethered to plastic holders with integrated platinum wires for EFS and mounted in 30 ml vertical organ baths filled with Krebs solution at 37 °C gassed with carbogen (95% O_2_/5% CO_2_). Isometric force was measured with SG4-90 transducers (SWEMA, Sweden) connected to KWS 3073 bridge amplifiers (HBM, Germany) and a Powerlab 8/30 using the LabChart7.2 software (ADInstruments, Australia). Strips were positioned 1-2 mm in front of the platinum wires, pre-stretched to 2-3 mN and given 1 h to equilibrate. Only strips producing ≥1 mN force when exposed to 60 mM KCl were included in further experiments. After 20 min equilibration, illumination was performed with a 455-460 nm LED (3 W, Avonec, Germany) run by a LDD-700L current source (MeanWell, Taiwan) and controlled by the PowerLab. The LED light source was orthogonally positioned on the outside of the organ bath and illumination of the strip specimen was performed through the glass walling of the bath. Light intensity was assessed using a water-resistant photoresistor circuit (NSL-19M51, Luna optoelectronics, USA) within the water bath prior calibrated via the PM100A/S120C-sensor power meter (Thorlabs). Electrical stimulation was applied in trains of 50 pulses at 10 Hz from a Grass-S8 stimulator (Grass Technologies, USA). Voltage and width of subsequent trains were increased between 80-150 V and 1-10 ms until responses saturated. Carbachol (CCh, Sigma-Aldrich) was added cumulatively of increasing concentrations. CCh and KCl were added to the solution and washed out not before a clear peak was reached. Responses were measured as differences between maximum force after and before substance application. Responses to light and EFS were quantified as maximal force between initiation and 10 s after termination of the respective stimulus. Values from pairs of responses to specific stimulations were averaged. The relevance of electrical coupling between SMC via gap junctions for light-induced contractions was assessed before and 15 min after adding 30 µM carbenoxolone (CBX, C4790, Sigma-Aldrich) to the organ bath. The isometric force was calculated as described above, the time to peak from the start of the contraction to the peak.

### Ca^2+^ imaging

Stomachs of ChR2 mice were placed in ice-cold Ca^2+^-free-Tyrode solution (Table 1). Antral smooth muscle strips were carefully detached from the underlying mucosal layer and incubated at 4 °C to be used within 12 h. Strips were subsequently incubated in carbogen-gassed Krebs solution containing 10 µM X-Rhod-1 (X14210, ThermoFisher Scientific) supplemented with 1:1000 Pluronic F-127 (PowerLoad™ Concentrate, P10020, ThermoFisher Scientific) for 2 h at 37 °C. After dye loading, strips were pinned onto a dish containing gassed Krebs solution at 37 °C and were given at least 5 min of equilibration time. X-Rhod-1 excitation was performed with a 500-600 nm LED (LEDHub Omicron) through an 562/40 BrightLine HC excitation filter and reflected via a HC BS 593 dichroic mirror. X-Rhod-1 emission was captured through a 641/75 BrightLine HC emission filter using a Zyla camera (Andor, Oxford Instruments, UK) at a framerate of 50 Hz. ChR2 stimulation was performed with the blue LED (460 nm/3000 mW) with 6 mW/mm^2^ for 50 ms before and 15 min after adding 30 µM CBX to the dish.

Since effective suppression of artefacts induced by any kind of mechanical displacement is critical for effective spatiotemporal analysis, captured videos (all 1280 × 1080 pixels) were investigated via extensive offline analysis (see Figure S1 and S2 for detailed description). The recordings were split into individual videos (750 frames) showing the first 15 s after the light pulse. Motion of the tissue strips was registered or tracked numerically in each pixel of each video with sub-pixel resolution after pre-processing the videos (spatio-temporal filtering, contrast-enhancement) as described previously 41, 42. Using the tracked displacement data, the videos were numerically stabilized or warped (affine transformation), effectively inhibiting the motion of the strips in the videos (Figure S1). To reduce computation times, motion tracking (Farnebäck 43) and warping was performed on a graphic processing unit (GPU, Nvidia, GeForce RTX 2080 Ti). To ensure the consistent overlap of all individual segments of each strip throughout the videos, all video frames in both the pre- and post-CBX videos were (cross-) registered with respect to the first frame in the pre-CBX video (see Supplementary Figures 1, 2). After numerical motion stabilization (NMS), ROIs were extracted from disk-shaped (diameter 11 pixels) small sub-regions around a pixel for further analysis (optical traces in Figure 5C). Within each strip only areas with clear signals were manually chosen for further analysis (see also Figure S1D and S2B-C). The optical traces after NMS were defined as Ca^2+^ transients. Within these areas, histograms showing the distributions of peak amplitudes and peak timings were derived from individual pixels (approx. 10,000 - 250,000 pixels per strip, signals with amplitudes <100 intensity counts were discarded). For statistical analysis, the values (peak amplitude and peak time for each strip) were derived from the peak heights and timings of log-normal fits to the distributions of peak amplitudes and times (Figure 5D-F). ChR2 expression was quantified by determining the amount of eYFP positive pixels applying the following procedure to every strip individually: Within the same area analyzed for Ca^2+^ imaging, ten representative areas without eYFP expression as well as ten areas with clearly visible eYFP signals were manually chosen and each cluster's pixel intensity was averaged to obtain a mean negative as well as a mean positive intensity value. Finally, the average between both values was set as the cutoff value to separate the two regions. Pixels with intensities below this cutoff were classified as negative, pixels above as positive and the percentage of positive pixels per strip was taken for further evaluation.

### Pressure measurements of whole-stomachs

Explanted stomachs were kept in preparation buffer before being transferred to a 120 ml organ bath containing carbogen-gassed Krebs solution at 37 °C and tethered onto a central stage. Three red (630-640 nm-5 W, Avonec) and three blue LEDs were mounted onto the inside wall of the bath surrounding the organ in 120° angles to achieve a global, reasonably homogenous panoramic stimulation as a prerequisite for efficient pressure development. Additionally, two platinum wires were placed next to the central corpus region for EFS. A fine tube catheter was inserted via the esophagus, perfused with Krebs solution (1 ml/h) and connected to a SP844 pressure transducer (MEMSCAP, France). Signals were recorded using a bridge amp, a PowerLab 8/30 and LabChart8.1.12. Following 20 min of equilibration, stomachs were subsequently stimulated by 60 mM KCl, blue light, EFS (20 pulses at 10 Hz, intensity 80-140 V) and CCh. For analysis of intrinsic, spontaneous contractions, the final 2 min interval of the initial 20 min equilibration time was analyzed. Only pressure waves with a change in amplitude higher than 0.5 cmH2O were considered for evaluation and amplitudes occurring in this interval were averaged. Afterwards, stomachs were incubated in 50 µM methylene blue for 30 min and illuminated with red light (3 min, 0.3 mW/mm^2^ for each LED). Henceforth, organs were given rest for another 20 min and the aforementioned stimuli were applied again.

In an additional series of experiments, we compared responses to illumination before and after adding 1 µM tetrodotoxin (Tocris, United Kingdom) to the organ bath and applied EFS to verify efficient blocking of neurogenic responses in order to test for a potential contribution of enteric neuronal circuits to light-induced pressure generation of isolated stomachs.

### Visualization of light-stimulated food propulsion

The day before the experiments, the food was changed from standard Sniff rat/mouse maintenance pallets to DietGel93M (both Sniff Spezialdiäten, Germany). Two percent brilliant blue FCF food color (RUF Lebensmittelwerk, Germany) was added to the DietGel93M. On the next morning, animals were sacrificed and the stomachs were placed in the organ bath as described above, catheterized and perfused with Krebs buffer containing 2% brilliant blue at 2 ml/h. After 1 h of perfusion, the catheter was pulled out of the stomach and the esophageal stub was tightly ligated. From the beginning of perfusion on, the duodenal stub was imaged from outside the organ bath using a microscope camera (dnt digimicro scale, dnt, Germany). Recordings were made under resting conditions, stimulation by one min long trains of 2 s light pulses applied every 5 s and, finally, during stimulation with 10 µM CCh.

Food propulsion from the duodenal stub of isolated stomachs was quantified by a person who was blinded in regard to the genotype of the respective donor animal. For each 5 s interval of video footage, a binary rating was made on whether propulsion of food content had taken place or not.

### Histology and immunofluorescence staining

**Figure 1:**
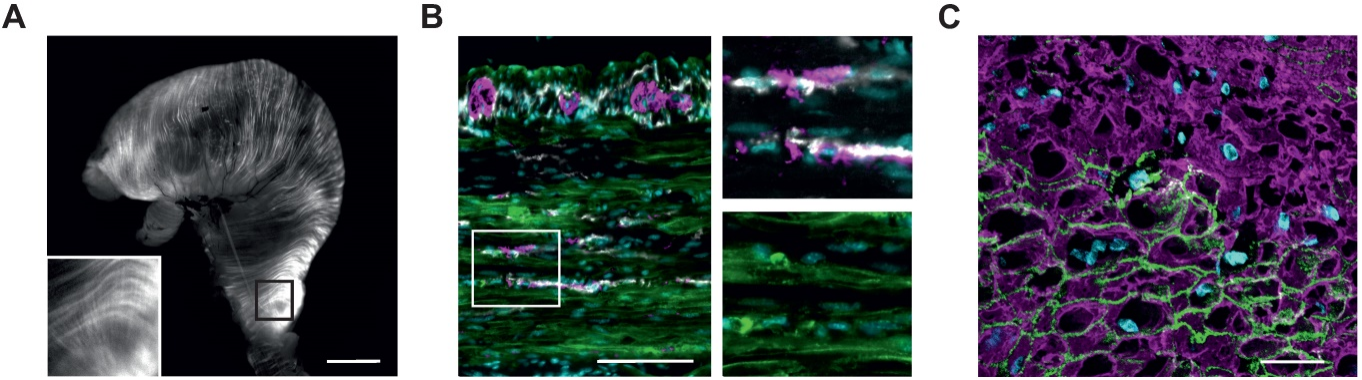
** Histological analysis of ChR2/eYFP expression in stomachs of transgenic animals. (A)** Explanted stomach of a ChR2 mouse with eYFP-fluorescence in circular and longitudinal orientation. Scale bar = 3 mm. **(B and C)** Tunica muscularis fluorescence images of the gastric antrum, pseudocolored with cyan for nuclei, green for ChR2/eYFP plus white for c-kit and purple for β-III-Tubulin (B, scale bar = 100 µm), and purple for α-smooth-muscle-actin. (C, Scale bar = 20 µm).

Stomachs were fixated in 4% formaldehyde for 24 h and then transferred to PBS containing 20% saccharose for 48 h. Organs were cryopreserved with Tissue-Tek (Sakura, Germany) and sectioned with a HM560 cryotome (Thermo Scientific, Germany) into slices of 8 µm thickness. Slices were permeabilised with 0.2% TritonX for 20 min and stained in PBS supplemented with 5% donkey serum for 2 h at room temperature with primary antibodies against either GFP (Sigma-Aldrich 11.814.460.001, 1:800), β-III-Tubulin (BioLegend 802001, 1:800) and c-Kit (Linaris MAK5302, 1:400) or against GFP (Chromotek 3H9-100, 1:400) and α-Smooth-Muscle-Actin (Sigma-Aldrich A2547, 1:400). Staining with secondary antibodies conjugated with Cy2 (1:200), Cy3 (1:400) or Cy5 (1:400) (JacksonLab, USA) diluted in PBS with 1:1000 DAPI was performed for 1 h at room temperature. Images of cryoslices were taken with an IX83 inverted fluorescence microscope (Olympus) equipped with an ORCA-flash4.0 digital camera (C11440, Hamamatsu Photonics, Japan) and the MT20 illumination system (Olympus) as light source. Acquisition of images was performed via the cellSens software with either a 20x objective (UPLSAPO20X, NA: 0.75, Figure 1B) or a 60x objective (UPLSAPO60X, NA: 1.35, Figure 1C) with following filter settings: 387/11 excitation, 410 beamsplitter and 440/40 emission for DAPI, 485/20, 504 and 525/30 for eYFP, 560/25, 582 and 607/36 for Cy3, and 650/13, 669 and 684/24 for Cy5.

For 3D confocal images, rectangular sections of the antral stomach wall underwent the PEGASOS clearing protocol as described previously 44. Samples were incubated with primary antibodies against GFP (1:200) and β-III-Tubulin (1:400) and subsequently with secondary antibodies conjugated with Cy2 (1:200) and Cy3 (1:400) for 5 days at 37 °C respectively. DAPI (1:1000) was added on the last day of secondary staining.

Whole-organ 3D images as well as images of isolated SMC were taken using a LSM 800 confocal microscope (Zeiss) equipped with spectral multi-alkali photomultiplier detectors. Z-stack acquisition was performed using a 63× objective (LCI Plan-Neofluar 63X, NA: 1.3) including a total view with a step size of 1.12 µm per slice via the ZEN 2.6 (blue edition) software (Zeiss) with a pinhole of 1 AU (53 µm for Cy3, 46 µm for Cy2 and 48 µm for DAPI) and the ZEN 2.6 auto z-correction option was used to increase laser power proportionally to tissue depth. Images of isolated SMC were taken with the 63x objective, a step size of 19 nm per slice and a pinhole of 1 AU (46 µm).

Whole-stomach fluorescence images were taken using a MVX10 macroscope (Olympus) equipped with an eYFP filter set (F46-003XL, AHF Analysetechnik) and a green LED (500-600 nm, LedHub, Omicron) coupled into the macroscope via a 2 mm light guide (NA 0.5).

### Statistics

Statistical data are shown as mean ± standard error of the mean and analyzed with the GraphPadPrism Software. Each dot either represents an independent experiment (number of each represented by n) for patch clamp, isometric force measurements and Ca^2+^ imaging or the stomach of one mouse (reported as N) for the intragastric pressure recordings and food propulsion analysis. We used an unpaired, two-tailed Student's t-test to compare ChR2 and control SMC. Comparison of normalized membrane resistance was performed via one-way ANOVA with Tukey's multiple comparison post-test. Isometric forces (unmodified as well as CBX-treated strips) and intragastric pressures (unmodified and TTX-treated organs) as well as time to peak values in CBX-treated strips were compared with two-way repeated measures ANOVA (within one mouse strain) and Sidak's multiple comparison to test the effect of ChR2 vs wild type control and Tukey's multiple comparison to test between different treatments. Comparison of propulsion probability was done using a two-way ANOVA and Sidak's multiple comparison to test the effect of ChR2 vs wild type control and Tukey's multiple comparison to test between different treatments. Intragastric pressures of methylene blue lesioned samples were compared with one-way repeated measures ANOVA and Tukey's multiple comparison test. Average amplitude and time to peak in Ca^2+^ imaging experiments were compared using paired, two-tailed Student's t-tests. p < 0.05 was considered statistically significant and significances are indicated as * for p ≤ 0.05, ** p ≤ 0.01, *** p ≤ 0.001 and **** p ≤ 0.0001.

## Results

### Mouse model

**Figure 2:**
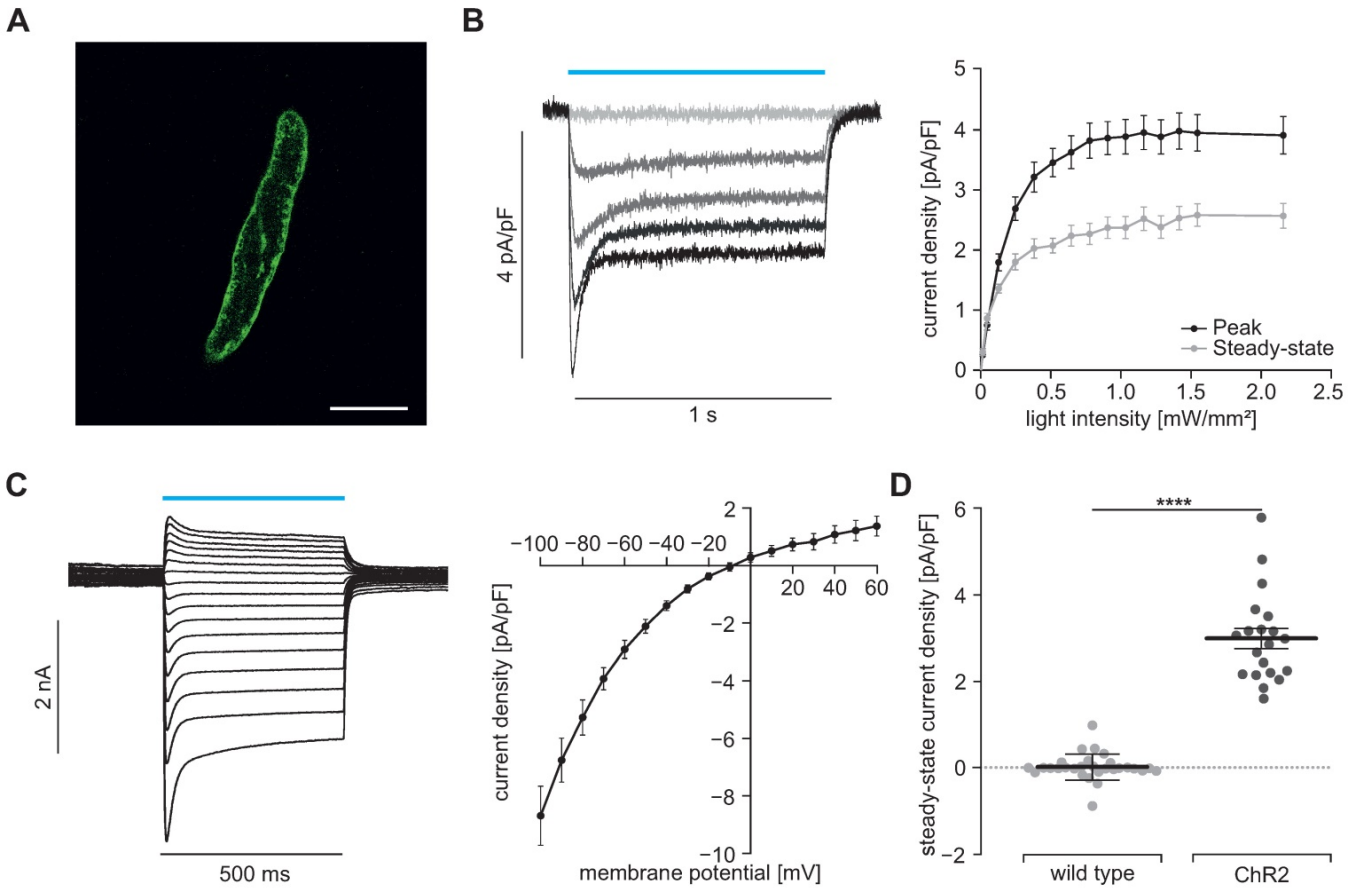
** Light-induced currents in SMC. (A)** Isolated SMC with membrane-bound ChR2/eYFP signal shown in green. Scale bar = 20 µm. **(B)** Representative traces (left) showing ChR2 currents at -60 mV induced by increasing light intensities (blue bar, from gray to black: 0, 0.05, 0.13, 0.25 and 0.7 mW/mm^2^) and analysis of peak and steady state currents (right, N = 4, n = 20). **(C)** Representatives examples and analysis of current-voltage-relation of steady state currents from -100 to +60 mV at 7.5 mW/mm^2^. **(D)** Comparison of steady state current density induced by supramaximal light (1 s, 7.5 mW/mm^2^) in ChR2 SMC (N = 4; n = 20) and SMC isolated from wildtype controls (N = 3; n = 25) evoked at a holding potential of -60 mV (p < 0.0001). Statistical analysis was performed with unpaired, two-tailed student's t-tests.

We used a previously described mouse model expressing ChR2 H134R fused to the fluorescence reporter eYFP under control of the chicken-β-actin promotor, which provides high expression rates in muscle cells 31. The explanted stomach showed elongated eYFP fluorescence signals, longitudinally and circularly arranged at the outer surface of the whole stomach (Figure 1A). Histological analysis in a tissue-cleared block of the antrum revealed that the eYFP signals were restricted to the circular and longitudinal muscle layers interspersed by the enteric nervous system and ICC (Figure 1B and Movie S1). Furthermore, eYFP fluorescence signals were restricted to the membranes of α-smooth muscle actin positive SMC (Figure 1C). After dissociation of the muscle layers, this membrane bound fluorescence signal could be found in 36±3% (n=5) of SMC (Figure 2A). Taken together, ChR2 expression is restricted to SMC, albeit at a rather low expression rate.

### Patch clamp experiments

**Figure 3:**
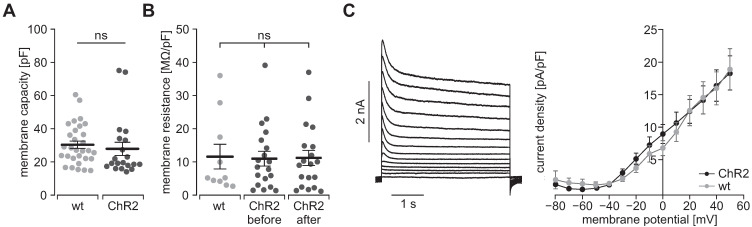
** Electrophysiological properties of ChR2/eYFP expressing (black) and control SMC from CD1 wild type mice (gray). (A)** Analysis of the cell size in whole-cell patch clamp experiments (N = 3, n = 30 for WT and N = 4, n = 20 for ChR2; p = 0.56, unpaired, two-tailed student's t-tests). **(B)** Comparison of membrane resistance normalized to membrane capacity (N = 4, n = 10 for WT and N = 5, n = 19 for ChR2; all p ≥ 0.98, 1-way ANOVA with Tukey's multiple comparison test). **(C)** Representative traces of a ChR2 cell (left) and analysis of voltage activated outward currents (right, N = 3, n = 10 for ChR2 and N = 3, n = 12 for WT, all p values ≥ 0.20, unpaired, two-tailed student's t-tests).

To prove the function of ChR2, we performed patch clamp experiments with freshly isolated SMC. Illumination with blue light (460 nm) induced inward currents in eYFP positive SMC which had the typical properties known from ChR2 H134R 45, 46: The strong initial inward current, called peak current, quickly desensitizes to a steady state current that remains stable for the duration of the illumination (Figure 2B). Varying the light intensity allows to control the amount of light-induced current with the half-maximal effective light intensity of the peak current being 0.2±0.01 mW/mm^2^ (n=20) and thus in the non-toxic area 47. Moreover, light-induced currents showed the typical inward rectification of ChR2 (Figure 2C) with the reversal potential at 0 mV proving the non-selectivity for cations. Importantly, SMC isolated from CD1 wild type mice did not react to illumination (Figure 2D). To exclude side effects by sole overexpression of ChR2, we analyzed the membrane capacity indicating the cell size (Figure 3A) as well as the membrane resistance before and after the illumination protocol (Figure 3B) and both did not reveal any differences. Furthermore, voltage-activated outward-directed currents elucidated by increasing voltage steps from -80 mV to +50 mV were similar at all tested steps (Figure 3C). These results demonstrate that ChR2 enables light-induced currents to control the membrane potential with high spatial and temporal precision without altering the electrophysiological properties of SMC.

### Isometric force measurements

**Figure 4:**
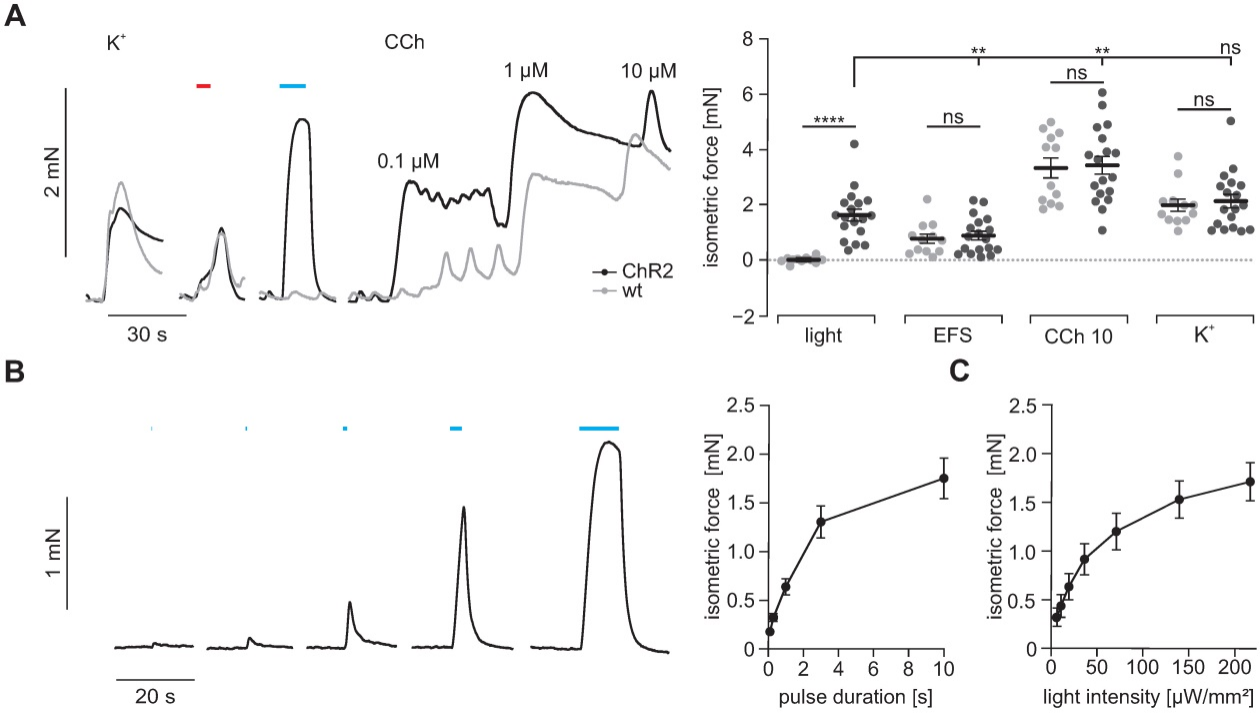
** Isometric force measurements of antral smooth muscle strips. (A)** Representative traces (left) and aggregated data (right) of isometric force measurements of gastric antrum strips from wildtype controls (N = 5, n = 12; in gray) and ChR2 animals (N = 6, n = 18; in black). Contractions were induced by 60 mM K^+^, EFS (red bar, 5 s, 10 Hz, 1-10 ms, 80-150V), blue light (blue bar, 10 s, 0.2 mW/mm^2^) and increasing concentrations of CCh (10 µM for statistical analysis). Statistical analysis was performed with 2-way repeated measures ANOVA with Sidak's multiple comparison test between ChR2 vs WT (p < 0.0001 for Light; p ≥ 0.97 for EFS, CCh and K^+^) and Tukey's multiple comparison test between different stimuli in ChR2 strips (p ≤ 0.003 for Light vs EFS and CCh; p = 0.38 for Light vs K^+^). **(B)** Representative example (left, shown are from left to right 0.1, 0.3, 1, 3 and 10 s long light pulses) and analysis (right) showing the effect of increasing pulse duration of light pulses with 0.2 mW/mm^2^. **(C)** Analysis of light intensity - isometric force relationship for 5 s long light pulses. B and C: N = 6; n = 20.

To prove that light-induced currents are sufficient to trigger contractions in SMC, we next measured isometric force generation of antral muscle strips upon illumination with blue light. Supramaximal light pulses triggered contractions only in ChR2 positive strips. Light-induced force generation was comparable to global depolarization by 60 mM K^+^ in the bathing solution indicating that light was able to completely activate all SMC despite an expression rate of ~36%. In clear contrast, neurogenic contractions elicited by supramaximal EFS were significantly weaker than those evoked by illumination. This can be explained by the co-stimulation of inhibitory nerves occurring with EFS 48. Contractions evoked by 10 µM CCh exceeded those elicited by illumination. This may be due to the fact that CCh at this concentration acts via two synergistic pathways, first by boosting ICC-mediated depolarization and activation of VDCC in SMC, second by directly triggering Ca^2+^ release and Ca^2+^ sensitization via protein kinase C and Rho-kinase 49, 50. Importantly, force generation upon stimulation with high [K^+^], EFS and CCh were similar in antral strips from ChR2 and wild type controls (Figure 4A) which excludes severe adverse effects of ChR2 overexpression to the contractile apparatus of SMC. Interestingly, the force of light-induced contractions was depending on the pulse duration (Figure 4B) and applied light intensity (Figure 4C). This ability to precisely control the amount of light-induced force production cannot be achieved by EFS. This data shows that ChR2-mediated depolarization can trigger contractions in SMC with high efficiency and precision.

### Analysis of electrical coupling in between SMC

**Figure 5:**
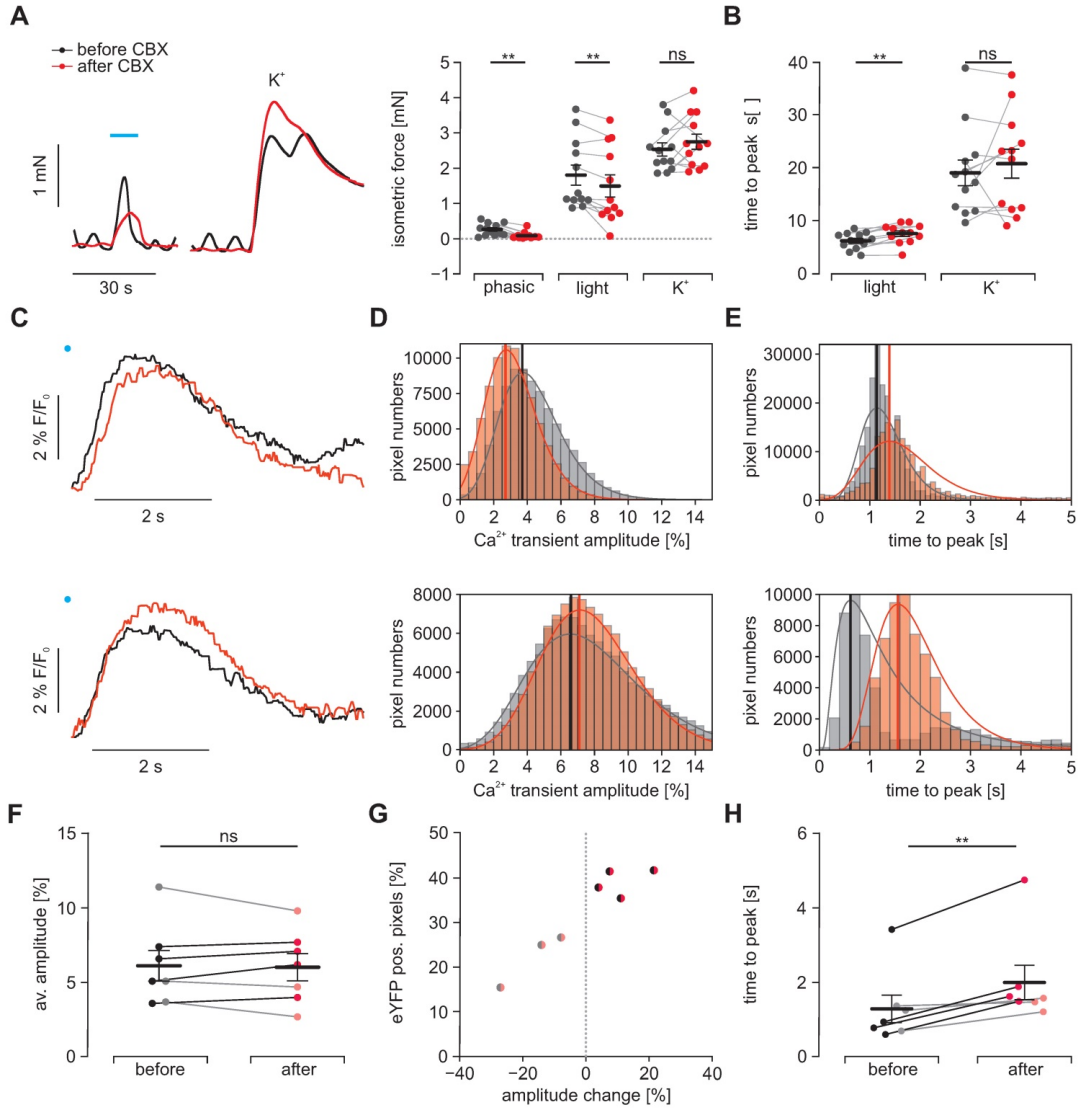
** Effects of electrical coupling via gap junctions.** To block electrical coupling via connexins, we applied 30 µM carbenoxolone (CBX, red) for at least 15 min in gastric antrum strips from ChR2 animals. **(A)** Representative traces (left) and aggregated data (right, N = 4, n = 12) of isometric force measurements. Contractions were spontaneous-phasic, induced by blue light (blue bar, 10 s, 0.2 mW/mm^2^) or 60 mM K^+^. Statistical analysis was performed with 2-way repeated measures ANOVA with Sidak's multiple comparison test (p = 0.003 for Phasic, p = 0.008 for Light, p = 0.54 for K^+^). **(B)** Analysis of time to peak (N = 4, n = 12). Statistical analysis was performed with 2- way repeated measures ANOVA with Sidak's multiple comparison test (p = 0.003 for Light; p = 0.63 for K^+^). C-H. Macroscopic Ca^2+^ imaging of antral smooth muscle strips following stimulation with blue light (11 mm^2^, 50 ms, 6 mW/mm²). **(C)** Original traces of tracked ROIs before (black) and after (red) CBX. Illumination indicated by blue dot. In the upper example, note the decreased amplitude and the prolonged time to peak, in contrast to the lower example with increased amplitude but also prolonged time to peak. **(D)** Histograms of peak heights (indicated by vertical line) of log-normal fits for light-induced Ca^2+^ transient amplitudes (fractional intensity change) of two different strips before and after CBX. Note the overall decrease in amplitude in the upper, and the increase in the lower example. **(E)** Histograms of peak times (indicated by vertical line) of log-normal fits for the analysis of the time interval between the light pulse and peak. Note the time prolongation in both examples. **(F)** Comparison of the light-induced average Ca^2+^ transient amplitude before and after CBX. Strips with an increasing amplitude are color coded in black/dark red and strips with a decrease in gray/light red. **(G)** Relationship between the fraction of eYFP positive pixels within the analyzed smooth muscle strip and the change in average amplitude. **(H)** Comparison of the average time to peak before and after CBX (p = 0.004). In F-H each dot represents a separately analyzed smooth muscle strip (n = 7). Statistical analysis was performed with paired student's t-tests.

To prove that light triggered activation spreads from ChR2 positive SMC to neighboring negative SMC, we blocked electrical coupling via gap junctions by applying CBX. In isometric force measurements, we observed a complete block of the intrinsic spontaneous phasic activity pattern indicating that the applied concentration of 30 µM effectively blocked electrical coupling from ICC to SMC. Regarding light-induced contractions, we found that CBX significantly diminished their maximum force and - even more effectively - prolonged the phase of contraction generation. Both findings fit well with the effects that would be expected when intercellular coupling between ChR2 positive and negative SMC are reduced by CBX. Importantly the amplitude and time to peak of contractions evoked by high [K^+^], which simultaneously activates each individual SMC irrespective of ChR2 expression, was unaltered (Figure 5A and B). To further investigate this effect in detail, we performed macroscopic Ca^2+^ imaging with antral smooth muscle strips (Figures S1 and S2 and Movies S2-5). Here, we found two different populations on a cellular scale as well as in the whole strip. The first population reacted to CBX with a delayed peak of the Ca^2+^ transient whose amplitude was also smaller. In contrast, the second population had almost simultaneous upstrokes with a higher and later peak (Figure 5C-E). Thus, we could not find a significant difference in the overall change in Ca^2+^ transient height (Figure 5F). However, when we classified the strips into these two populations, we found that the ones with overall delayed and diminished Ca^2+^ transients had less eYFP positive areas indicating a lower ChR2 expression rate within the imaged area (Figure 5G). Both populations showed a delay in the time to peak which was more pronounced in the second population with likely higher ChR2 expression and less current spread to ChR2 negative cells (Figure 5F). In part, this more efficient activation of ChR2 positive cells will counteract the impaired activation of ChR2 negative SMC.

Taken together, isometric force measurements and Ca^2+^ imaging suggest that light-induced stimulation of ChR2 positive SMC is activating neighboring non-expressing SMC and that at least in part electrical coupling via gap junctions is involved.

### Intragastric pressure measurements

**Figure 6:**
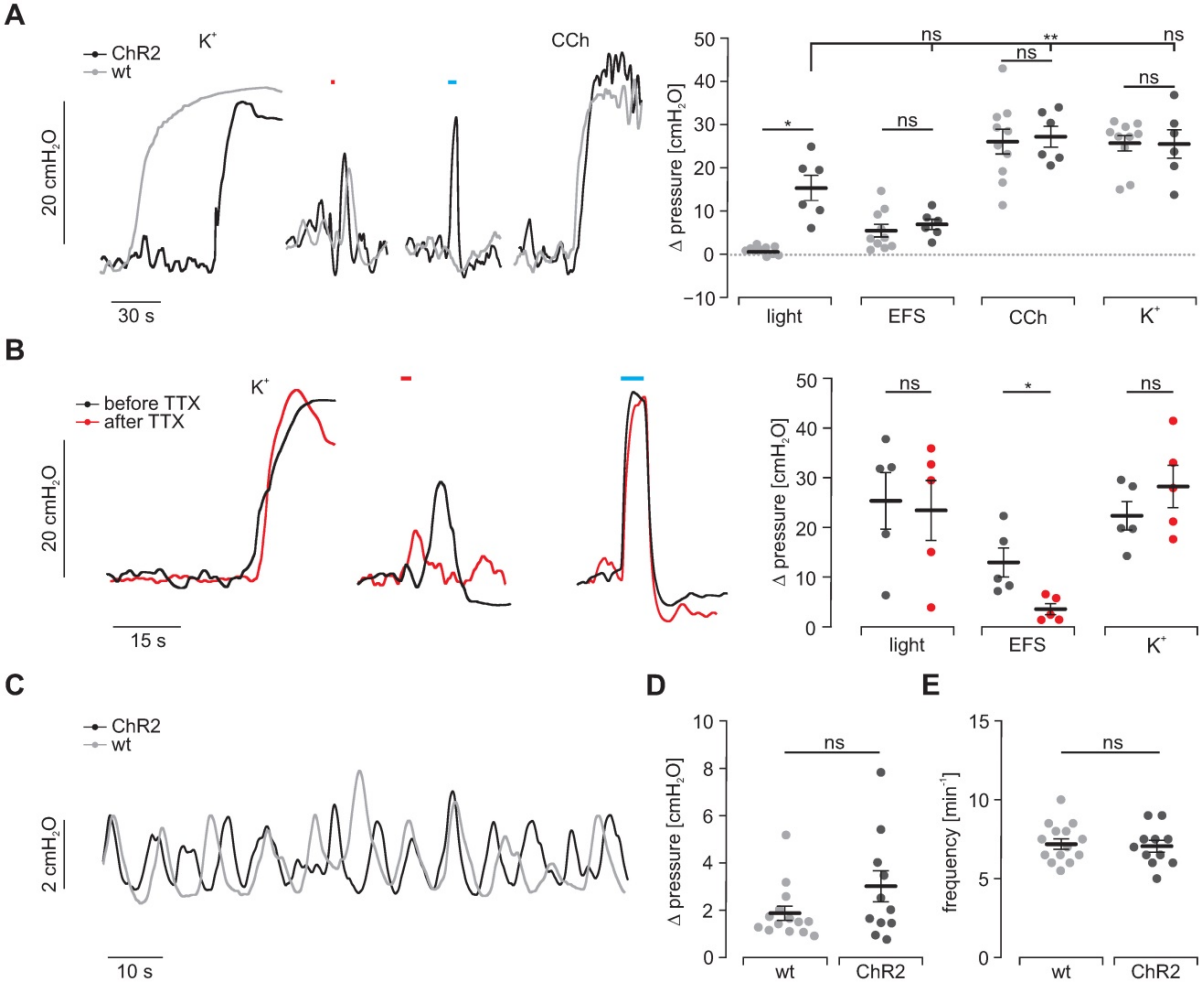
** Intragastric measurement of pressure upon illumination in intact stomachs. (A)** Representative traces (left) and aggregated data (right) of intragastric pressure measurements from wildtype controls (N = 10; in gray) and ChR2 animals (N = 6; in black). Contractions were induced by 60 mM K^+^, EFS (red bar, 2 s, 20 Hz, 1-10 ms, 80-140V), blue light (blue bar, 5 s, 0.2 mW/mm^2^) and 10 µM CCh. Statistical analysis was performed with 2-way repeated measures ANOVA with Sidak's multiple comparison test between ChR2 vs WT (p = 0.01 for Light; p ≥ 0.73 for EFS, CCh and K^+^) and Tukey's multiple comparison test between different stimuli in ChR2 stomachs (p = 0.001 for Light vs CCh; p = 0.052 for Light vs EFS or K^+^). **(B)** Representative traces (left) and aggregated data (right) of intragastric pressure measurements from ChR2 animals (N = 5) before (black) and after (red) adding 1 µM TTX. Contractions were induced as described in A. Statistical analysis was performed with 2-way repeated measures ANOVA with Sidak's multiple comparison test (p = 0.14 for Light, p = 0.03 for EFS, p = 0.68 for K^+^). **(C)** Representative traces of spontaneous contraction patterns in intact stomachs of ChR2 transgenic (black) and wildtype mice (gray). **(D and E)** Comparison of pressure amplitudes (N = 11, p = 0.15) and frequency (N = 14, p = 0.63) of spontaneous contractions within the final 2 min of the initial 20 min equilibration time. Statistical analysis was performed with unpaired, two-tailed student's t-tests.

To understand the physiological significance of light-induced contractility, we assessed the generation of intragastric pressure in the intact stomach. Three LEDs were arranged at 120° angles around intact stomachs. Maximal illumination triggered peak pressures similar to those elicited by EFS but smaller compared to the ones induced by 60 mM K^+^ and CCh (Figure 6A). Importantly, responses to CCh, 60 mM K^+^ and EFS were again non-distinguishable between intact stomachs of ChR2-expressing and CD1 wild type animals (Figure 6A). To exclude any potential contribution of the enteric (e.g. mechanosensitive) neuronal circuits 51 on light-induced contractions, we blocked neuronal activity by applying tetrodotoxin and found a significant reduction of force trigged by EFS but not by light or high [K^+^] (Figure 6B). Furthermore, spontaneous activity patterns did not differ in rate as well as height between both groups (Figure 6C-E). This proves homogenous and concerted activation of SMC necessary for grinding and propelling of food contents towards the duodenum since non-effective illumination or inhomogeneous expression of ChR2 would cause escape-movements of non-contracted wall-segments and thus prevent pressure increases.

### Visualization of light-stimulated food propulsion

**Figure 7:**
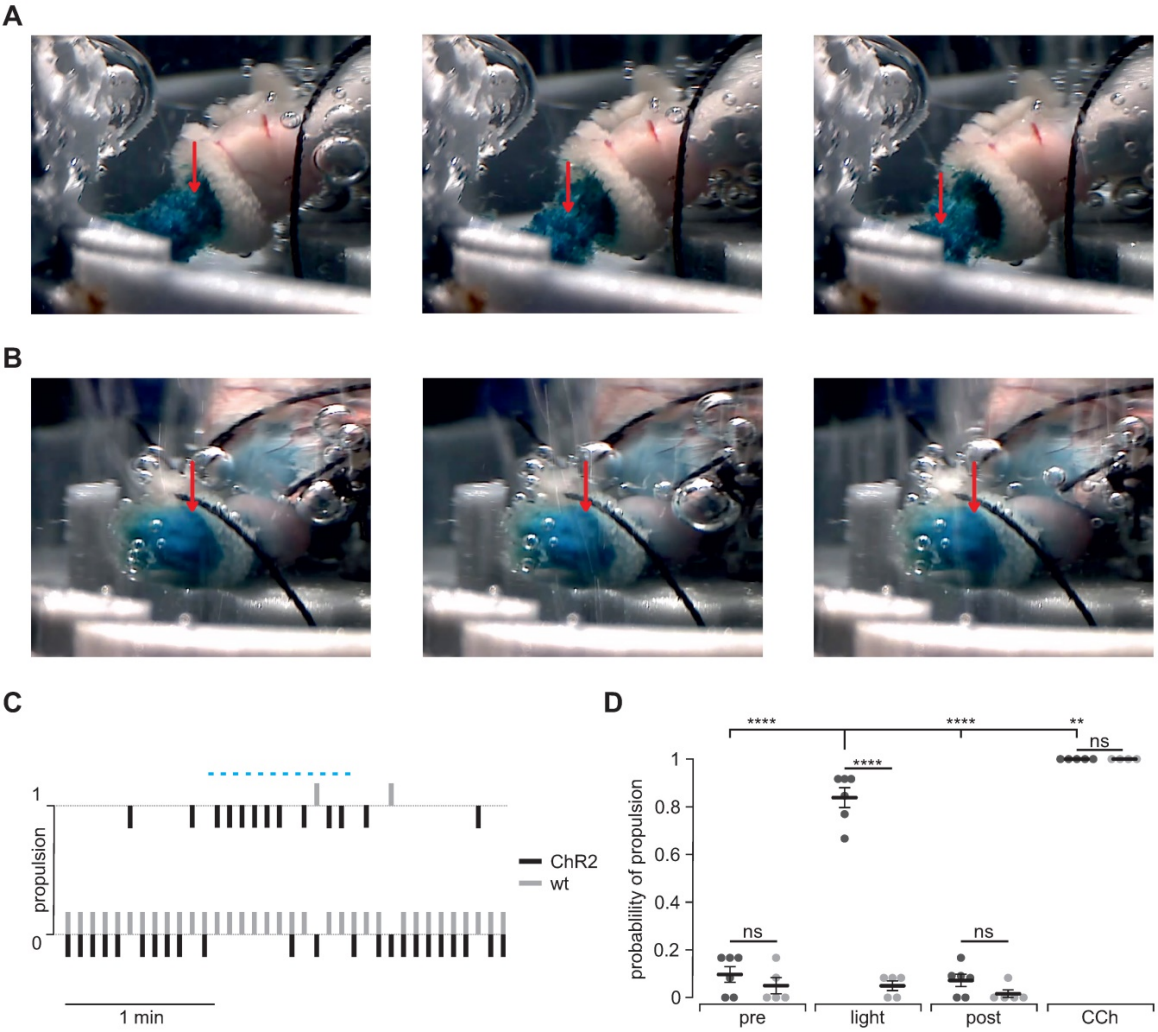
** Propulsion and gastric emptying upon illumination in intact stomachs. (A and B)** Image series of ChR2 (A) and WT (B) intact stomachs. Shown images are chosen from 5 s intervals each and directly after a respective 2 s light stimulus. Note the obvious food propulsion in A which cannot be observed in B. **(C)** Representative traces showing the analysis of food propulsion over time for a ChR2 (black) and a wild type stomach (gray) before, during and after the light stimulation protocol (blue bar, 12 times 2 s, 0.2 mW/mm^2^). 0 equals no detectable propulsion, while 1 represents obvious food propulsion as shown in A. Analysis was performed by a blinded person. **(D)** Aggregated data showing the probability of propulsion before, during and after light stimulation (N = 6 for ChR2, N = 5 for WT), and the application of 10 µM CCh (N = 5 for ChR2, N = 4 for WT). Statistical analysis was performed with 2-way ANOVA with Sidak's multiple comparison test between ChR2 vs WT (p < 0.0001 for Light; p ≥ 0.52 for Pre, Post and CCh) and Tukey's multiple comparison test between different stimuli in ChR2 stomachs (p< 0.0001 for Light vs Pre or Post; p = 0.001 for Light vs CCh).

To prove that optogenetic stimulation is able to enforce gastric motility and emptying, we quantified light-induced aboral food propulsion via the duodenal stub during patterned light stimulation in ChR2 expressing (Figure 7A) and wild type control stomachs (Figure 7B). In ChR2 expressing stomachs, we could detect light-induced food propulsion that was restricted to the illumination period (Figure 7C). Importantly, light did not have any effects in wild type control stomachs whereas CCh was efficient in both groups (Figure 7C and D).

### Gastroparesis model

**Figure 8:**
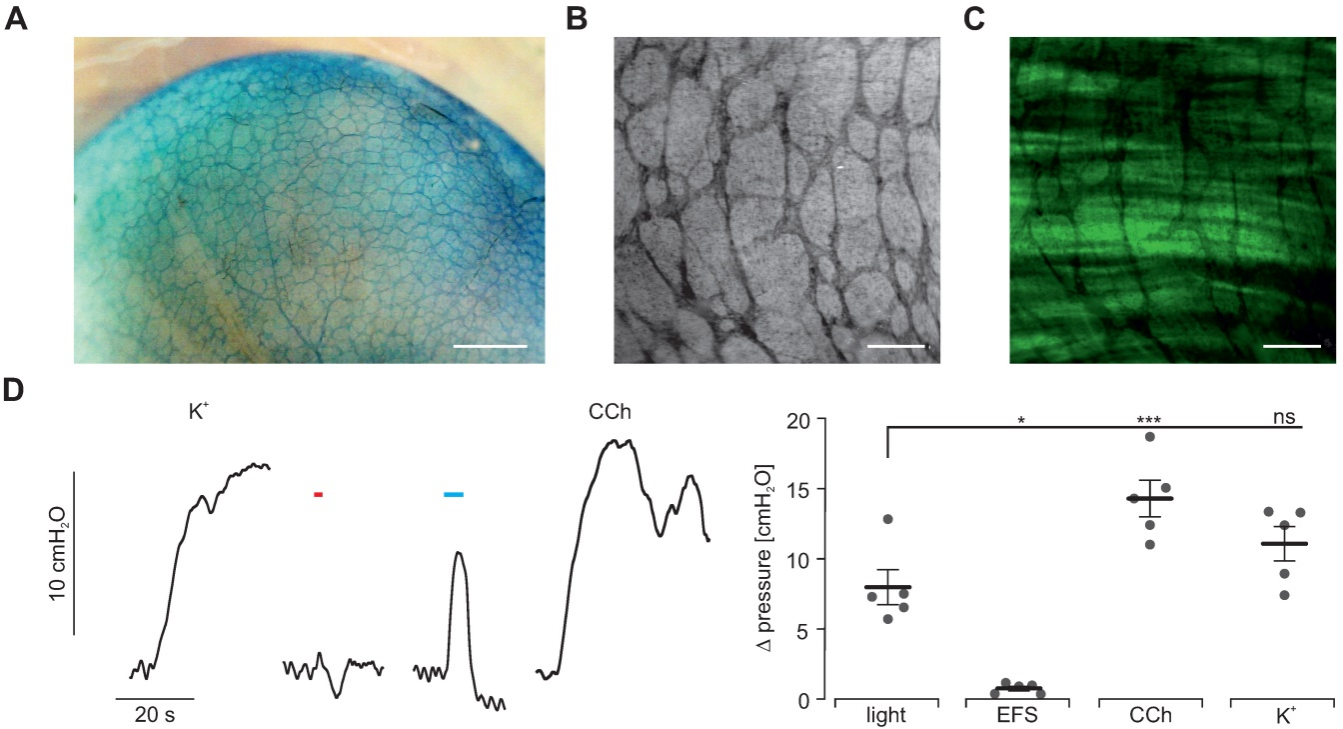
** Methylene blue-based photodynamic lesioning in whole-organ stomach preparations. (A)** Image of an explanted stomach of a ChR2 transgenic mouse after 30 min incubation with 50 µM methylene blue. Scale bar = 1 mm. **(B and C)** Close-up view of methylene blue-staining (B) and depiction of native eYFP fluorescence (C) in an explanted stomach of a ChR2 transgenic mouse demonstrating methylene blue specificity to nerval fibres and ICC but not smooth muscle tissue. Scale bar = 200 µm. **(D)** Representative traces (left) and statistical analysis (right) of whole-organ intragastric pressure measurements of ChR2 transgenic animals (N = 5) after methylene blue photodynamic lesioning. Contractions were induced by 60 mM K^+^, EFS (red bar, 2 s, 10 Hz, 1-10 ms, 80-140V), blue light (blue bar, 5 s, 0.2 mW/mm^2^) and 10 µM of CCh. Statistical analysis was performed with 1-way repeated measurements ANOVA and Tukey's multiple comparison test (p ≤ 0.02 for Light vs EFS or CCh; p = 0.17 for light vs K^+^).

To demonstrate that light-stimulation of SMC is sufficient to trigger contractions even when ICC and neuronal function is impaired, we took advantage of photodynamic lesioning of ICC and neurons using the photosensitizer methylene blue 52. After 30 min long incubation, methylene blue was detectable within the enteric nervous system (Figure 8A) spreading into smooth muscle layers but not affecting the SMC and ChR2 expression (Figure 8B-C). Next, we used red illumination to induce methylene blue-mediated phototoxicity. Afterwards, spontaneous activity was abolished and EFS completely failed to trigger contractions proving the destruction of ICC and neurons. In clear contrast, illumination with blue light still induced intragastric pressure albeit being less pronounced than before the photolesion. However, the pressure increases induced by CCh and K^+^ application stimuli were reduced in a similar range (Figure 8D). Thus, we conclude that the function of ICC and enteric motoneurons was completely lost whereas SMC were only slightly affected. Importantly, direct optogenetic stimulation of SMC was still able to induce contractions and significant pressure waves.

## Discussion

In this study, two lines of evidence indicate that direct optogenetic stimulation of SMC can be used to control gastric contractility: morphologically, by proving the selective expression of ChR2 in SMC using advanced histological analysis and functionally, by demonstrating the persistence of effective light stimulation after disrupting the activity of ICCs and neurons using selective photo lesioning and pharmacological inhibition.

Physiologically, electrotonic current spread from ICC to SMC is considered to be the final step of intrinsic gastrointestinal pacemaking. This electromechanical coupling between ICC and SMC is tightly controlled by neurochemical signaling, e.g. by acetylcholine-binding to muscarinic receptors 53, 54. If ICC spread sufficient current to depolarize the SMC above the threshold for VDCC, Ca^2+^ increases which induces contraction 55. Our characterization of light-induced currents in ChR2 expressing SMC, the resulting Ca^2+^ transients and isometric force measurements prove that these currents are sufficient to reach the threshold potential of VDCC. Given the multitude of molecular and cellular mechanisms that have been suggested to be relevant for gastric smooth muscle contractility 56, it is an essential finding of this study that selective depolarization of SMC is sufficient to trigger contractions. In the transgenic animal model only one third of the gastric SMC did express ChR2/eYFP. This can be explained by the fact that the mice were generated by electroporation and random integration of the linearized DNA into the genome of the embryonic stem cells 31. This random integration might have led to different integration sites which can be silenced or not in dependence of the cell cycle, maturation, age and subtype of each SMC. However, we also observed clustering of the ChR2/eYFP positive cells which hints at clonal proliferation. It is well known from gastric and vascular SMC that various subpopulations of SMC exist within one functional syncytium which has been detected by electrophysiological behavior, functional analysis, actin isoforms and gene expression 57-62.

Interestingly, stimulating only this third of all SMC is sufficient to induce contraction amplitudes in the same range as a uniform depolarization of all SMC by 60 mM K^+^. This finding underlines the efficiency of electrical coupling between adjacent SMC and indicates that ChR2-mediated currents are far more than required to reach the VDCC threshold in one SMC. Our analysis of SMC electrical coupling via isometric force measurements and macroscopic Ca^2+^ imaging proves the superiority of optogenetic stimulation to quantify SMC coupling due to its temporal precision. These experiments prove the relevance of connexins in electrical coupling responsible for the spread of light-induced activation from ChR2 positive SMC to neighboring non-expressing SMC. Taking advantage of the yet unprecedented possibility to selectively stimulate SMC using optogenetics, we were able to reveal differences between ICC-SMC heterocellular and SMC-SMC homocellular coupling: The same concentration of CBX which completely abolished spontaneous activity deriving from ICC had still significant, but less pronounced effects on light-induced force generation and Ca^2+^ transients. This hints at additional coupling mechanisms which might be responsible for SMC-SMC coupling, as they have already been suggested on the basis of indirect evidence from biophysical modelling and ultrastructural studies, but yet without experimental foundation 63-65. This highlights the power of optogenetics to investigate electrical coupling and define the process of electrical recruitment of gastric smooth muscle in great detail due to the unique combination of fine-tunable expression and the spatial as well as temporal precision of illumination. Importantly, we could find no signs of side-effects by ChR2/eYFP expression which is in line with reports from other muscle tissue 31,32,66,67. However, we cannot exclude that subtle phenotypic differences might have escaped our attention and we did not perform experiments with animals expressing only the fluorescence protein.

The therapeutical potential of any approach for the treatment of gastroparesis depends on its efficiency despite the underlying pathologic conditions. Direct optogenetic stimulation of SMC can shortcut the network of ICC completely and is thus unsusceptible against pathological alterations of ICC and enteric neurons themselves and their connectivity towards SMC. In addition, optogenetic stimulation can be performed pain-free, with less side effects and a better control on the amount of force generation compared to EFS. We suggest the feasibility of direct optogenetic stimulation of SMC in cases of gastroparesis by demonstrating light-induced pressure generation within the stomach after methylene blue induced phototoxicity. The fact that methylene blue is taken up selectively by ICC and neurons of various intestinal tissues allows selective photo lesioning with red light 52,68. We adapted this approach for isolated stomachs. To assure effective impairment of ICC and neurons, we had to accept slight alterations of SMC function indicated by reduced contractions triggered by exogenous high K^+^ concentration or CCh. Still, blue light induced significant pressure increases which were in a similar range compared to EFS before photo lesioning. Although the spatiotemporal pattern of antral contractions induced by the pulsed panoramic illumination in our experiments may still differ from that of intrinsic peristaltic waves, we were nevertheless able to demonstrate an efficient triggering of food transport.

Interestingly, wireless control of implanted LED devices has already been successfully applied within the colon of transgenic mice to increase the fecal pellet output 28 and recently in the bladder to trigger urination 69. The SMC layer on the outer surface of the human stomach is only approximately 2 mm thick 70,71. This is important since it allows the use of blue light for efficient illumination of the whole muscular layer with reasonable light intensities and thus also more restricted light effects assuming similar light absorption of the SMC tissue compared to the heart 72. Therefore the recently described expandable integrated optoelectronic mash with µLEDs allows quasi panoramic illumination 69 which could be applied simultaneously but also sequentially to simulate physiological contractility waves. On the other hand, the thinness of the SMC layers allows the use of blue light activated ChR variants although red light can be applied with much higher energies without toxic effects 26. The best suited ChR variant can thus be freely chosen and will be determined by the optimal kinetics but also less pronounced inward rectification 46 and thus more effective depolarization to activate Ca^2+^ channels. An alternative to cation selective ChRs could be Cl^-^ selective ones 73-76. However, these channels depend on the reversal potential for Cl^-^ ions which is probably lower than the membrane potentials which can be reached by cation selective ChRs 77,78.

Gene transfer to express ChR2 has been proven to be safe in the eye by adenoassociated viruses (AAV) and is currently in the phase I/II of clinical testing 47. At first sight, AAV-based gene transfer resulting in comparable ChR expression rate to our transgenic animal model seems to be feasible. AAV subtypes with high transduction rates of SMC have been described as well as SMC specific promotors 79-82. Since the SMC layer is at the outside of the stomach and thin, gene painting is the most promising approach to obtain high and localized ChR expression. However, it will be challenging to find an approach for specific expression in gastric SMC without SMC within in the gastric arterioles. A sophisticated approach would be to couple the expression of ChR to the differential expression patterns of “tonic” (i.e. vascular) and phasic (i.e. distal gastric and intestinal) smooth muscle cells 83. Importantly, the rate of phasic contractions of the human stomach is rather low (ca. 3/min) so that the time in between stimuli would be sufficient to allow blood supply even if light induced co-contractions of intramural vessels occurred.

Furthermore, the immune response against the combination of the various parts required for this treatment have to be carefully characterized and prevented 26,84. Importantly, we herein deliver only a proof-of-concept for the mere feasibility to control gastric contractions as well as motility *ex vivo*. Many technical optimizations have to be achieved before optogenetic gastric pacemaking can be suggested as therapeutical option.

Taken together, direct optogenetic stimulation of SMC allows for control of gastric contractility with unprecedented spatial and temporal precision. Therefore, optogenetic stimulation is a method that could help to improve existing therapeutic strategies by elucidating the physiology and pathophysiology of gastric motility and could itself become a tantalizing new option for the treatment of gastroparesis.

## Supplementary Material

Supplementary tables and figures.

Supplementary movie S1.

Supplementary movie S2.

Supplementary movie S3.

Supplementary movie S4.

Supplementary movie S5.

## Figures and Tables

**Table 1:** Table indicating the single components of the used solutions for storage of stomachs, digestion of smooth muscle cells, patch clamp experiments and preparation as well as performance of isometric force and intragastric pressure measurements

	Tyrode solution	Digestion mix	External solution	Internal solution	Preparation buffer	Krebs solution
NaCl	135	135	140		145	112
KCl	5	5	5.4	50	4.5	4.7
CaCl_2_			1.8		1	2.5
MgCl_2_	1	1	2	1		1.2
K-Aspartat				80		
Mg-ATP				3		
Glucose	10	10	10			11.5
EGTA				10		
EDTA					0.025	
MgSO_4_					1.2	
NaH_2_PO_4_					1.2	
KH_2_PO_4_						1.2
NaHCO_3_						25
Hepes	10	10	10	10	5	
pH	7.4	7.4	7.4	7.2	7.4	
pH adjustment	NaOH	NaOH	NaOH	KOH	NaOH	

## Abbreviations

ANOVA: analysis of variance
CBX: carbenoxolone
CCh: carbachol
ChR2: channelrhodopsin2
CR: cross registration
EFS: electric field stimulation
eYFP: enhanced yellow fluorescent protein
ICC: interstitial cells of Cajal
ROI: region of interest
SMC: smooth muscle cells
TTX: tetrodotoxin
VDCC: voltage-dependent calcium channels
WT: wild type
