# Supplementary material for: Direct optogenetic stimulation of smooth muscle cells to control gastric contractility
Source: Theranostics. 2021 Mar 20;11(11):5569–84. doi: 10.7150/thno.53883 (PMC8039938; doi:10.7150/thno.53883)
Supplement: Supplementary file 1 — Supplementary tables and figures. [file thnov11p5569s1.pdf]

Supplementary figures and tables:

Direct optogenetic stimulation of smooth muscle cells to control gastric contractility

Markus Vogt, Benjamin Schulz, Ahmed Wagdi, Jan Lebert, Gijsbert J. van Belle, Jan Christoph, Tobias Bruegmann* and Robert Patejdl *

Table 1: Table indicating the single components of the used solutions for storage of stomachs, digestion of smooth muscle cells, patch clamp experiments and preparation as well as performance of isometric force and intragastric pressure measurements.

	Tyrode solution	Digestion mix	External solution	Internal solution	Preparation buffer	Krebs solution
NaCl	135	135	140		145	112
KCl	5	5	5.4	50	4.5	4.7
$CaCl_2$			1.8		1	2.5
$MgCl_2$	1	1	2	1		1.2
K-Aspartat				80		
Mg-ATP				3		
Glucose	10	10	10			11.5
EGTA				10		
EDTA					0.025	
$MgSO_4$					1.2	
NaH_2PO_4					1.2	
KH_2PO_4						1.2
$NaHCO_3$						25
Hepes	10	10	10	10	5	
pH	7.4	7.4	7.4	7.2	7.4	
pH adjustment	NaOH	NaOH	NaOH	KOH	NaOH	

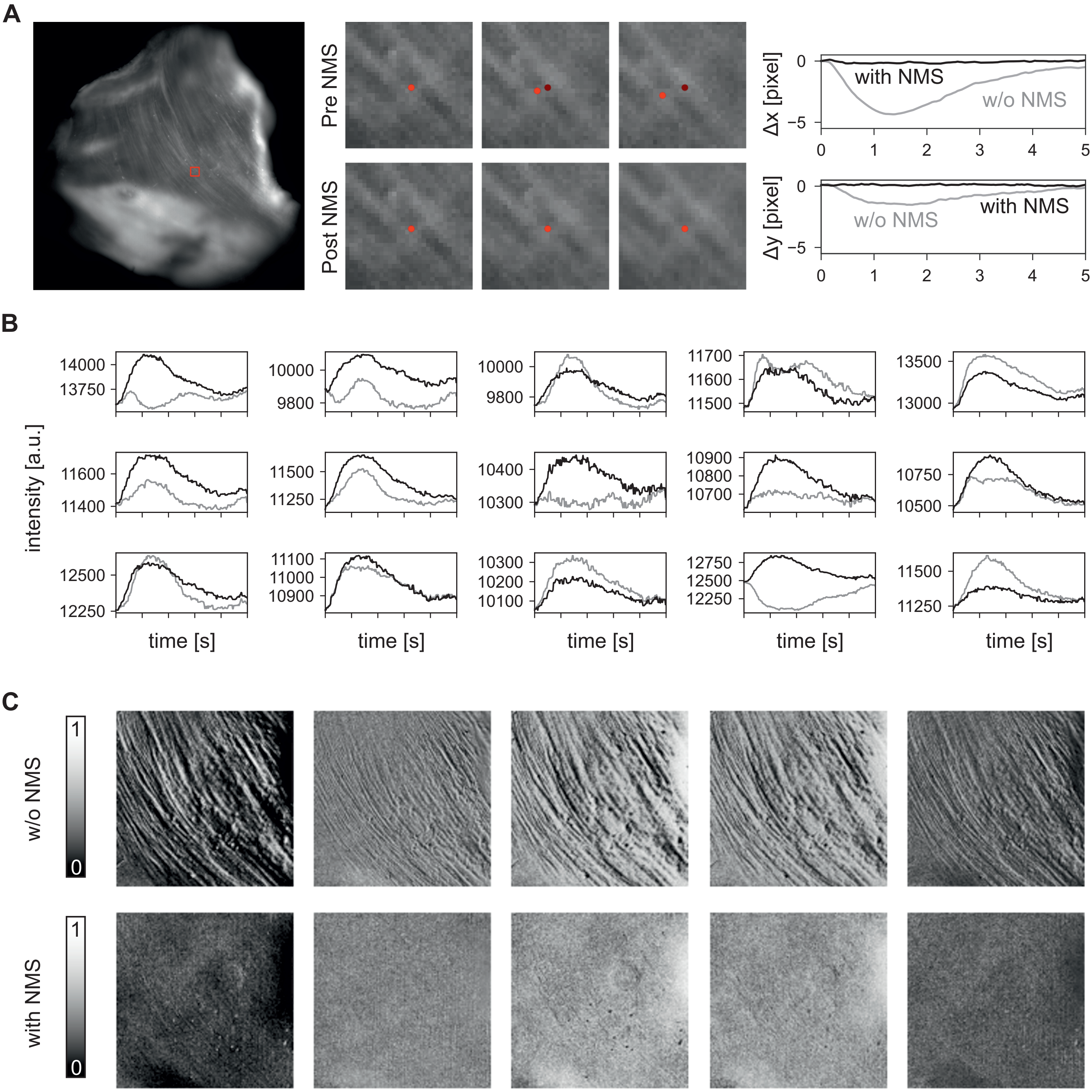

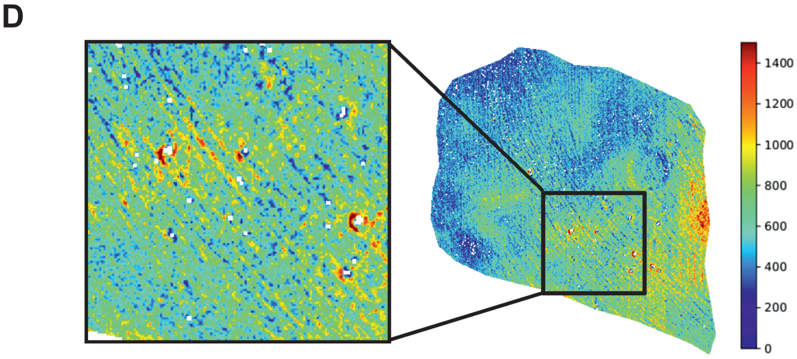

Supplementary Figure 1. Numerical motion-stabilization (NMS) and post-processing of video data obtained during Ca^{2+} imaging. **A.** Left: Antral SMC strip during Ca^{2+} imaging. The red square indicates the area shown in the panels in the middle and its analysis on the right. Center Top: Close up of area within red rectangle on the left, c.f. Movies S2 and S3. Motion (towards left, downwards) of a single tissue segment or pixel (red dot) in the original video before NMS at 0.0 sec., 0.6 sec. and 1.8 sec determined with numerical motion tracking (with respect to original position in first frame of the video = dark red dot). Center Bottom: Same time points from the video after NMS, accordingly the tissue segment remains at the center of the field of view throughout the video sequence (red dot). Right: Motion of tissue segment in x- and y-directions over time before (gray) and after (black) NMS. **B.** Optical traces extracted from small disk-shaped ROIs (diameter 11 pixels) without (w/o, gray) and with (black) NMS. Note the varying direction of signal change before NMS due to motion artifacts, which are removed after NMS. **C.** Reduction in motion artifacts in optical maps during Ca^{2+} imaging of the central part of the SMC strip due to numerical motion-stabilization (NMS). Top: Pictures at representative time points from a video pre (w/o) NMS after numerically amplifying and visualizing temporal intensity changes (pixel-wise normalization [0,1]). Spatial patterns are largely caused by motion (optical flow, see also [41]). Bottom: Pictures from the same time points as top from the video with NMS with substantially reduced motion artefacts and homogenous intensity increases (white) and decreases (black) during the Ca^{2+} transient. **D.** Heat Map displaying the absolute peak amplitude for each pixel within an area of analysis (c.f. Supplementary Figure 2B) as indicated by the color code.

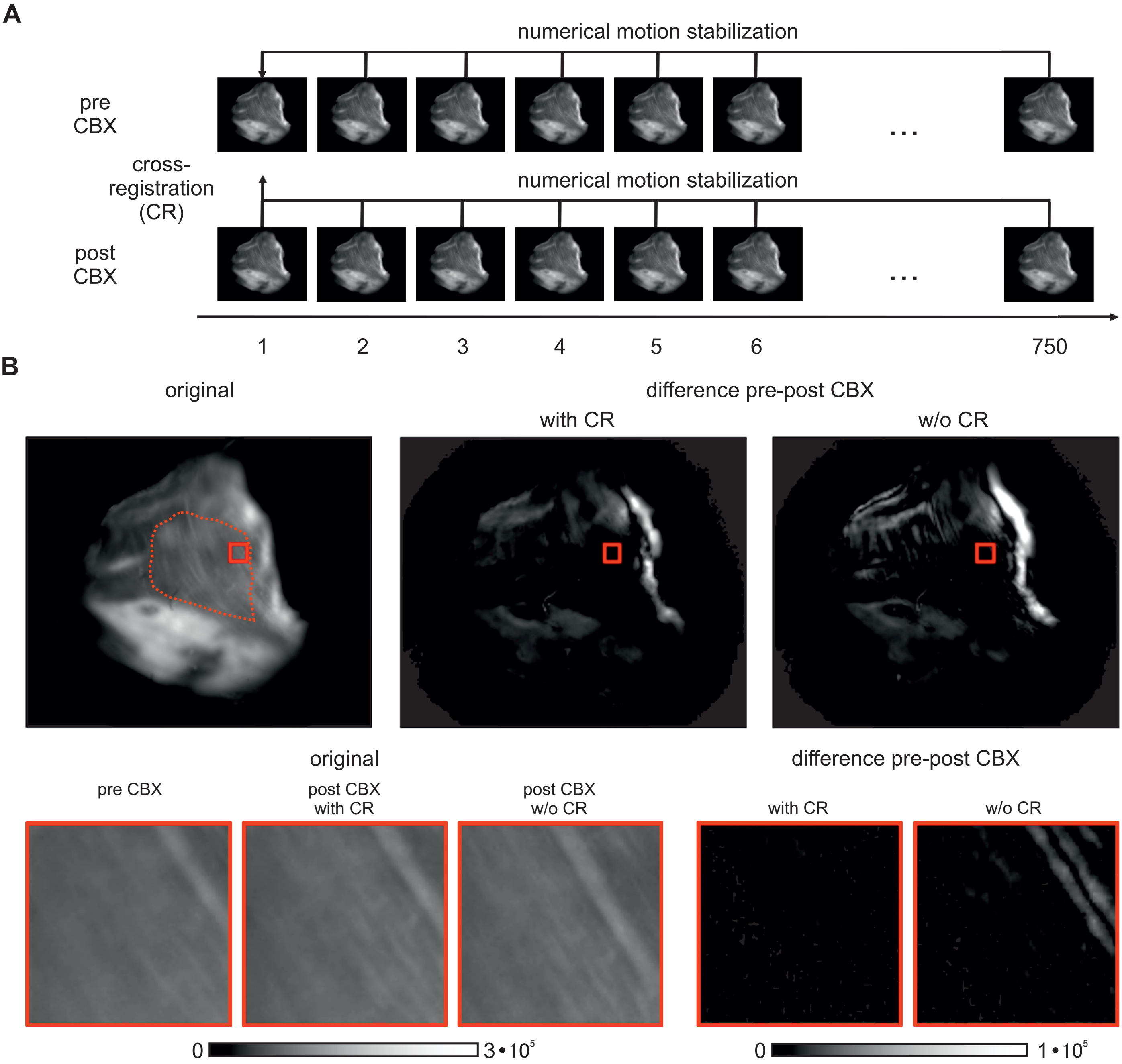

Supplementary Figure 2. Numerical tracking and motion compensation scheme consisting of numerical motion tracking and -stabilization and cross-registration (CR). **A.** All video frames (1-750) in both the pre- (Top) and post-CBX (Bottom) videos were (cross-) registered (i.e. tracked and warped) with respect to the first frame in the pre-CBX video to ensure the consistent overlap of all individual segments of each strip throughout the videos and in between measurements (15 min apart). **B.** Effect of cross-registering all video frames of post-CBX video onto first frame of pre-CBX video. Top: First image of the whole sequence with the dashed line indicating the area for final analysis, chosen manually because this area includes fewest movement artefacts. Right: Difference without (w/o) and with CR. Bottom: Original frames of the area indicated by red rectangle in the top row. Without CR, there are significant mismatches between the two videos (pre- and post-CBX). Accordingly, the absolute differences computed in each pixel between the two videos become smaller only after CR and NMS.

Movie S1. Different perspectives onto a 3D antral gastric tissue block (200 x 90 µm) after tissue clearing with PEGASOS method preserving the native fluorescence signal. Nuclear staining is shown in blue, ChR2/eYFP fluorescence in green and β-III-Tubulin in magenta.

Movie S2. Motion tracking of an antral SMC strip during Ca^{2+} imaging. Left: Original video before application of CBX (pre-CBX). Motion of tissue segments (in the range of 0-10 pixels) analyzed with numerical motion tracking indicated by red, blue, green and yellow dots. Original positions indicated by darker dots. Motion tracking data is available for every pixel. Right: The same video as left after numerical motion stabilization (NMS). Motion in the central part of the strip is effectively inhibited (dots indicate output from tracking of the warped, motion-stabilized video). Analyses of the motion tracking and NMS of the same area can be found in Figure S1A and Movie S3. Video is shown at 2.5x speed.

Movie S3. Numerical motion stabilization (NMS) with sub-pixel precision (field of view from red rectangle in Figure S1A, close-up view of same strip as in Movie S2). Left: Motion of a single tissue segment or pixel (red dot) in original video before application of CBX (pre-CBX) determined with numerical motion tracking (with respect to original position = dark red dot). Motion tracking data is available for every pixel. Right: motion-stabilized video, in which all video frames are deformed (warped) to match the configuration of the tissue in the first frame of the pre-CBX video. Analyses of the motion tracking and NMS of the same area can be found in Figure S1A and Movie S2. Video is shown at 2.5x speed.

Movie S4. Reduction in motion artifacts in optical maps obtained during Ca^{2+} imaging of an antral SMC strip due to numerical motion-stabilization (NMS). Left: Central part of stomach strip in original video without NMS after numerically amplifying and visualizing temporal intensity changes (pixel-wise normalization [0,1]). Spatial patterns largely caused by motion (optical flow, see also [41]). Right: Same part of stomach strip in video after NMS and after numerically amplifying and visualizing temporal intensity changes (pixel-wise normalization [0,1]). Motion artifacts are substantially reduced and intensity increases (white) and decreases (black) are largely caused by the tissue's local intensity in- and decrease during the Ca^{2+} transient, see also Figure S1C. Video is shown at 2.5x speed.

Movie S5. Ca^{2+} imaging video from an antral SMC strip prior (left) and after (right) numerical motion stabilization. Difference video (pixels in frame t+10 frames were subtracted from pixels in frame t to highlight temporal intensity changes) superimposed onto original grayscale video (green: positive intensity changes, violet: negative intensity changes, linearly encoded from -500 to 500 a.u.). Video is shown at original speed.
